# Can the cortical bone trajectory screw technique be an alternative method to the pedicle screw in posterior lumbar fusion? A systematic review and meta-analysis

**DOI:** 10.5152/j.aott.2021.21169

**Published:** 2021-11-01

**Authors:** Kun-Tae Kim, Myung-Geun Song, Eun-Chang Lee, Min-Seok Seo, Dong-Yeong Lee, Dong-Hee Kim

**Affiliations:** 1 Department of Orthopedic Surgery, Gyeongsang National University Hospital, Regional Trauma Center, Jinju, Republic of Korea; 2 Department of Orthopedic Surgery and Institute of Health Science, Gyeongsang National University School of Medicine, Gyeongsang National University Hospital, Jinju, Republic of Korea; 3 Clinic of Orthopedic Surgery, Barun Hospital, Jinju, Republic of Korea

**Keywords:** Cortical bone trajectory, Pedicle screw, Posterior lumbar fusion, Meta-analysis, Systematic review

## Abstract

**Objective:**

The aim of this study was to verify the practicability of the cortical bone trajectory (CBT) method by comparing the clinical outcomes including the complications between the CBT method and pedicle screws (PSs).

**Methods:**

MEDLINE, EMBASE, Cochrane Central Register of Controlled Trials (CENTRAL), web of Science, and SCOPUS electronic databases were searched for relevant articles published through March 2021 that compared the outcomes of the CBT and PSs. The data search, extraction, analysis, and quality assessment were performed according to the Cochrane Collaboration guidelines. The clinical and radiological outcomes of both techniques were evaluated using various outcome measures.

**Results:**

Sixteen studies with a total of 1173 patients were included in the study. The outcomes in the meta-analysis indicated that the use of CBT fixation showed better results for overall complications (*P* < 0.0001), symptomatic adjacent segment disease (sASD) (*P* = 0.007), superior facet joint violation (SFJV) rate (*P* = 0.007), operating time (*P* = 0.007), intraoperative blood loss (*P* < 0.00001), incision length (*P* = 0.002), length of hospital stay (*P* = 0.0006), and revision rates (*P* = 0.02). However, there were no statistically significant differences in fusion rates or detailed complications including hardware complications, wound infections (all *P* > 0.05) between the CBT method and PS fixation groups.

**Conclusions:**

The present study revealed that the CBT method was associated with higher functional recovery, lower surgical morbidity rates, lower revision rates, and lower overall complication rates including sASD and SFJV rates. However, both the CBT method and PSs had similar fusion rates, complications including hardware complications (screw malposition, screw loosening, and screw pullout) and wound infections. Thus, the CBT method did not outperform the PSs in all aspects. Therefore, it is recommended to select a surgical method in consideration of the patient’s bone mineral density, the condition of the pars interarticularis, or the skill level of the surgeon. Prognostic evaluation through long-term follow-up is required, and more high-quality randomized controlled trials are required to verify and strengthen our results.

**Level of Evidence:**

Level III, Therapeutic Study

## Introduction

Since Boucher^1^ described the pedicle screw (PS) fixation method, it has been adopted as the gold standard of posterior lumbar fusion, thanks to its good biomechanical stability, rigid fixation, and high fusion rate.^2-^^4^ However, correction loss and low fusion rates due to screw loosening were observed in patients with osteoporosis.^5^ To increase securing of the bone by pedicle screws, Santoni et al.^6^ proposed a new cortical trajectory, reinforcing the cortical bone contact, regardless of the bone mineral density (BMD). The screw corridor, defined from the medial to the lateral path in the transverse plane and caudal to the cephalad path in the sagittal plane, increases the security of the bone screw. The medial entry point of the cortical bone trajectory (CBT) screw has the advantages of minimal muscle damage and preservation of the superior facet joint. Several biomechanical studies showed increased pullout strength and insertional torque and better resistance in flexion and extension loading with the CBT construct.^7-^^9^ A randomized prospective trial study^10^ revealed lower surgical morbidity rates, and comparative studies^11–13^ showed better outcomes with CBT methods than with PS techniques.

However, other studies reported that PSs had better fatigue resistance in poor bone quality and stiffness in lateral bending than the CBT method, with stability equivalent to that of CBT screws.^14–17^ Also, the CBT technique has not always shown superior clinical outcomes including operative time and complication rates^18^ and might be technically difficult for inexperienced surgeons.^19^ Although the advantages of CBT have been demonstrated, there is insufficient definitive proof, such as in vivo analyses and surgical contraindications, to confirm it as an acceptable substitute for the PS method.^20^ Therefore, we performed a systematic review and meta-analysis to verify the feasibility of the CBT method by comparing various outcomes and complications between the CBT method and PSs.

## Materials and Methods

### Search strategy and study selection

We used multiple comprehensive databases to find literature that compared the outcomes of PSs and CBT screw fixation. This study was based on the Cochrane methods of review, and reporting was in accordance with the Preferred Reporting Items for Systematic Reviews and Meta-Analyses (PRISMA) statement. To identify the relevant studies, we used the controlled vocabulary and free text words described in Appendix 1 to search the MEDLINE, EMBASE, the Cochrane Central Register of Controlled Trials, Web of Science, and the SCOPUS databases. We attempted to identify all relevant studies regardless of language, publication type (article, poster, conference paper, and instructional course lecture), publication journal, or publication year. The search was updated in March 2021 and included the reference lists of the studies and any review articles identified. Study inclusion was decided by two independent researchers in accordance with the selection criteria, and when it was difficult to evaluate the relevance of the subject after reading the titles and abstracts, the full article was perused.

### Inclusion criteria

Studies were included in this meta-analysis if: (1) they were randomized controlled trials (level I) or prospective and retrospective studies that compared CBT screw fixation with PS fixation in posterior lumbar fusion using the open technique; (2) the authors provided sufficient information regarding the incidence of complications, radiological outcomes, and complications; and (3) the comparison outcomes included at least one of the following: fusion rates, superior facet joint violation (SFJV) rates, screw malposition, operating time, intraoperative blood loss, incision length, length of hospital stay, revision surgery rates, symptomatic adjacent segment disease (sASD), and the incidence of wound infections.

### Exclusion criteria

Studies were excluded based on the following criteria: (1) noncomparative studies, single-arm studies only reporting the PS or the CBT method; (2) percutaneous fixation, robotic-assisted fixation, or in vitro (laboratory or biomechanical) studies; (3) systemic reviews, meta-analyses, case reports, editorials, letters, animal experiments, and cadaveric studies; and (4) an average follow-up period of less than 1 year.

### Data extraction

Two investigators independently recorded the following data based on a predefined data extraction form: (1) surgical techniques; (2) design of the study, the sample size of each group, age, and sex, follow-up period, and surgical technique; and (3) the comparison of the outcomes and complications. When the two investigators did not reach a consensus, the records were reviewed by a third investigator.

### Data collection and analysis

We independently assessed the titles or abstracts of the studies identified using the searching strategy and then reviewed the full papers for final inclusion. Uncertainties were resolved through discussion and consensus. We independently abstracted the eligible data onto predefined forms and checked them for accuracy. We also collected information on study characteristics, patient demographic data (Table 1), the results of studies including radiological outcomes and surgical feasibility (Table 2). Then, we determined the number of subjects and the means ± standard deviations (SD) of the demographic data and various outcomes in the two groups.

### Assessment of methodological quality

Two investigators independently assessed the methodological quality of each study using the Cochrane Risk of Bias Tool for randomized controlled trials (RCTs)^21^ and measured the inter-reviewer agreement for RCTs (Cohen’s k). For qualified analysis of the non-randomized controlled trials, the Newcastle−Ottawa assessment scale, a tool for evaluating clinical cohort studies was used. A maximum of nine stars was awarded on a total of 3 items, which were selection, comparability, and exposure, to assess the validity of the research. Any disagreement was resolved through discussion or following a review by a third investigator.

### Statistical analysis

The main purpose of this review was to compare the various outcomes between the groups of patients who underwent posterior lumbar fusion using the CBT or PS methods. To compare the surgical feasibility between the groups, we assessed various outcomes such as operation-related outcomes and complications. We used Review Manager ver. 5.3 (The Cochrane Collaboration, Oxford, UK) to estimate the overall pooled effect size for each outcome and conducted a meta-analysis of the included studies using a random-effects model. For the continuous outcomes (surgical time, intraoperative blood loss, and length of hospital stay), we calculated the mean difference (MD) with 95% confidence intervals (CIs) using an inverse variance method. For the dichotomous outcomes (complications and fusion rate), the risk ratio (RR) between the groups was calculated using the Mantel−Haenszel method. We assessed statistical heterogeneity among the studies using I-squared (I^2^) and chi-squared test (*P* values). The heterogeneity was considered significant when *P* < 0.1 or I^2^ > 50%.

## Results

### Identification of studies

**Figure 1.**
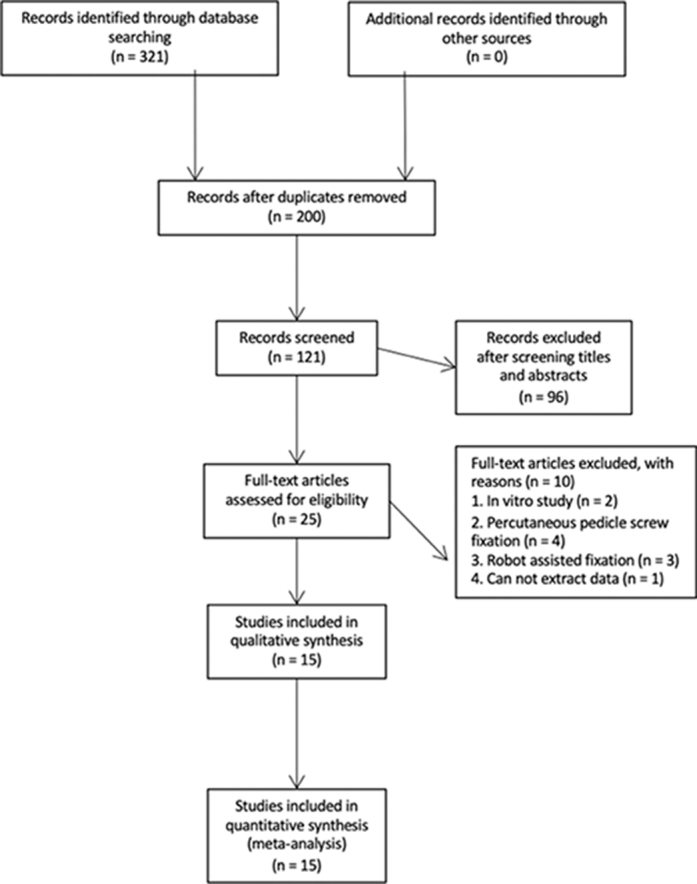
Flow diagram of study selection.

We initially identified a total of 321 relevant articles from MEDLINE (90), EMBASE (88), the Cochrane Library (14), Web of Science (101), and SCOPUS (28). Of these, 200 were duplicated in the databases. After screening the remaining 121 articles using titles and abstracts, we excluded 96 according to the exclusion criteria. Then, we excluded 10 articles following a thorough full-text review of all 25 articles according to the exclusion criteria. Finally, 15 studies^10–13,22–32^ were included for data extraction and meta-analysis (Figure 1). The outcomes between the patients in the CBT and PS groups and indication of CBT method are summarized in Tables 2 and 3.

### Quality and publication bias of the included studies

All 15 studies^10–13,22–32^ (two RCT, 13 cohort studies) were included in the meta-analysis, with a total of 1173 patients (591 patients in the CBT and 582 patients in the PS group). The risk of selection bias between the two groups was low. To evaluate the methodologic quality, the Cochrane Risk of Bias Tool was used for the RCTs. The included trials showed a low risk of bias, indicating that most studies were of good quality based on the current system. Fourteen cohort studies were assessed by the Newcastle−Ottawa scale (Table 4). No assessable confounding factors for evaluating the demographic data were found. The follow-up period was recorded, with longer periods associated with lower risk of bias. All 15 studies included in this meta-analysis had a low risk of selection bias and compared the demographic data of the subjects undergoing lumbar fusion surgery, with none assessing the possible confounding factors. Follow-up was defined as the interval between surgery and outcome evaluation.

### Overall Complications and Detailed Factors

**Figure 2.**
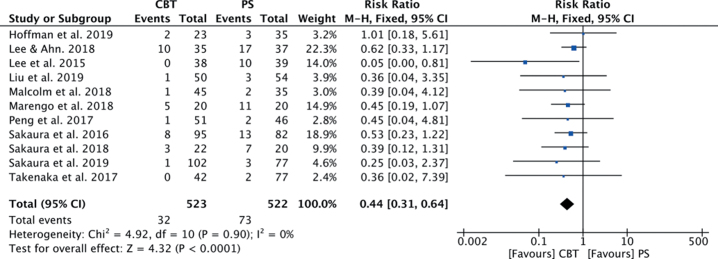
Overall complications.

The overall complication rates^10–13,25,26,28–32^ were analyzed in 11 studies. The CBT group showed lower overall complication rates [RR = 0.51, 95% CI (0.37 to 0.71), *P* < 0.0001; heterogeneity, (*P* = 0.73), *I*^2^ = 0%] than the PS group (Figure 2).

**Figure 3.**
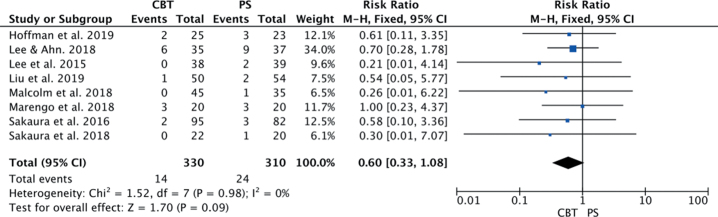
Hardware complications.

**Figure 4.**
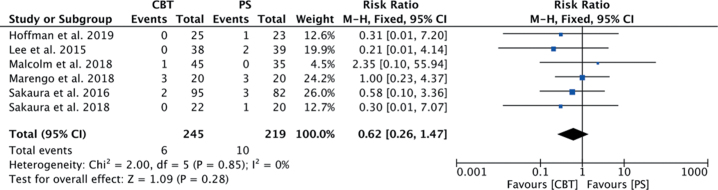
Screw malposition rates.

**Figure 5.**
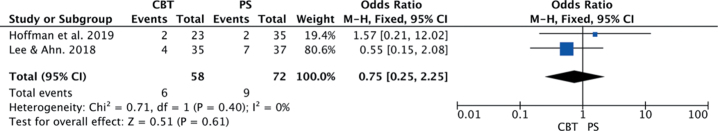
Screw loosening rates including screw pullout.

Eight studies^10–12,26,28–31^ reported hardware complications. There was no significant difference between the two groups [RR = 0.60, 95% CI (0.33 to 1.08), *P* < 0.09; heterogeneity, (*P* = 0.98), *I*^2^ = 0%] (Figure 3). Among the hardware complications, six studies^10–12,28–30^ showed screw malposition. However, the difference was not statistically significant [RR = 0.62, 95% CI (0.26 to 1.47), *P* < 0.28; heterogeneity, (*P* = 0.85), *I*^2^ = 0%] (Figure 4). Two studies^26,30^ reported screw loosening including screw pullout but there was no significant difference between the two groups [RR = 0.75, 95% CI (0.25 to 2.25), *P* < 0.61; heterogeneity, (*P* = 0.40), *I*^2^ = 0%] (Figure 5).

**Figure 6.**
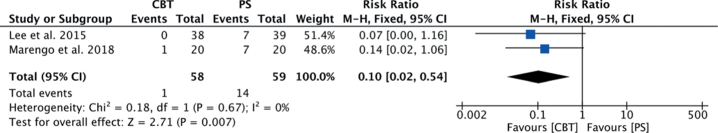
Superior facet joint violation rates.

**Figure 7.**
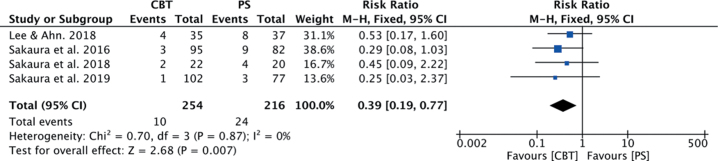
Symptomatic adjacent segment disease.

**Figure 8.**
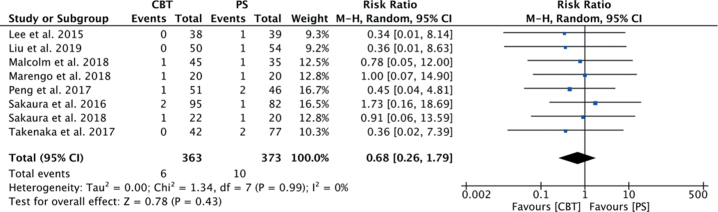
Wound infections.

Two studies^10,29^ showed SFJVs and there was a significant difference between the two groups [RR = 0.10, 95% CI (0.02 to 0.54), *P* = 0.007; heterogeneity, (*P* = 0.67), *I*^2^ = 0%] (Figure 6). Four studies^11,12,26,32^ revealed sASD, which was significantly higher in the CBT group than in the PS group [RR = 0.39, 95% CI (0.19 to 0.77); heterogeneity, *P* = 0.007, *I*^2^ = 0%] (Figure 7). Eight studies^10–13,25,28,29,31^ reported wound infections but there was no significant difference between the two groups [RR = 0.68, 95% CI (0.26 to 1.79), *P* = 0.43; heterogeneity, (*P* = 0.99), *I*^2^ = 0%] (Figure 8).

### Surgical practicability

**Figure 9.**
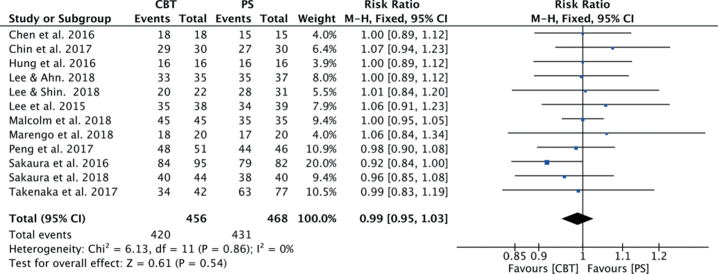
Fusion rates.

The fusion rates^10–13,22–29^ were analyzed in 12 studies. There was no significant difference between the two groups [RR = 0.99, 95% CI (0.95 to 1.02), *P* = 0.54; heterogeneity, (*P* < 0.00001), *I*^2^ = 97%] (Figure 9).

**Figure 10.**
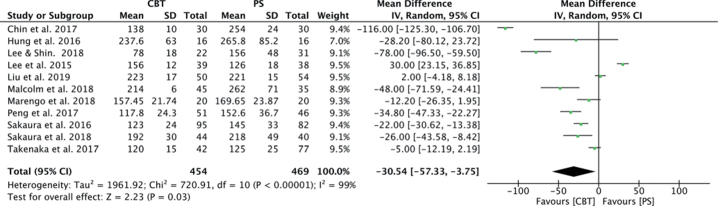
Operation time.

**Figure 11.**
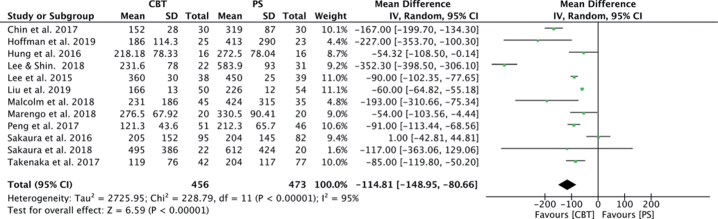
Intraoperative blood loss.

**Figure 12.**
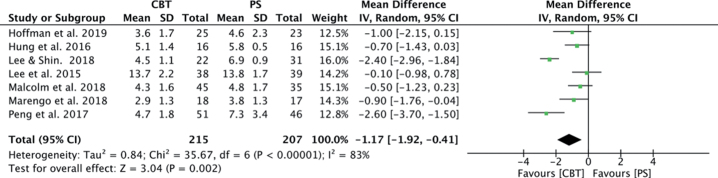
Incision length.

**Figure 13.**
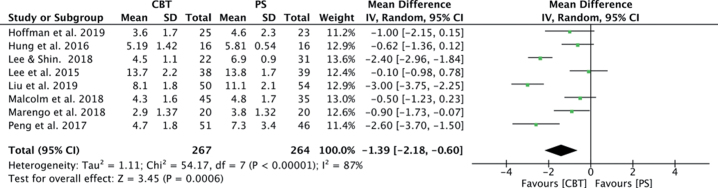
Length of hospital stay.

Operating time (Figure 10),^10–13,23–25,27–29,31^ intraoperative blood loss (Figure 11),^10–12,23–25,27–30^ incision length (Figure 12) ^10,23,25,27–30^and the length of hospital stay (Figure 13) ^10,23,25,27–30^ were analyzed. The CBT group showed shorter operating time, less intraoperative blood loss, shorter incision length, and shorter hospital stay than the PS group [(MD = −26.98; 95% CI (−46.70 to −7.26), *P* = 0.007); heterogeneity, (*P* < 0.00001), *I*^2^ = 99%], [(MD = −104.69; 95% CI (−136.19 to −73.18), *P* = < 0.00001); heterogeneity, (*P* < 0.00001), *I*^2^ = 95%], [(MD = −1.17; 95% CI (−1.92 to −0.41); *P* = 0.002); heterogeneity, (*P* < 0.00001), *I*^2^* *= 83%], [(MD = −1.39; 95% CI (−2.18 to −0.60); *P* = 0.0006); heterogeneity, (*P* < 0.00001), *I*^2^ = 87%], respectively.

**Figure 14.**
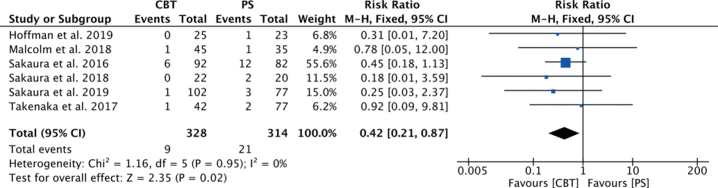
Revision rate.

Regarding revision surgery, five studies^11–13,28,30^ reported that the CBT group showed lower additional surgery rates than the PS group with statistical difference [RR = 0.42, 95% CI (0.21 to 0.87), *P* = 0.02; heterogeneity, (*P* = 0.95), *I*^2^ = 0%] (Figure 14).

## Discussion

Since the initial CBT technique^6^ was introduced for posterior lumbar fusion, studies^7,8,13,15,33–35^ have reported the biomechanical and radiological outcomes. Also, to confirm the benefits of CBT, comparative studies with PS have been reported. However,^20^ whether CBT can be used as an alternative to the PS method remains debatable because the results were mostly from biomechanical and clinical CBT studies.^36^ Thus, the purpose of this study was to reveal the feasibility of the CBT method by comparing the outcomes and complications between the CBT and PS methods.

The incidence of complications is a critical factor in estimating the superiority of the CBT or PS fixation method. The overall complication rates regarding hardware, SFJV, sASD, and wound infections were significantly higher in the PS groups. However, there was no significant difference in each complication rate including hardware complications and wound infections.

The types of hardware complications included screw malposition and screw loosening or screw pullout in both groups (Table 5). Screw malposition has uncertain clinical outcomes for the patient and can cause spinal cord injury or nerve root injury. Screw loosening or screw pullout can cause nonunion or early revision.

Screw malposition was the most common complication between the two groups without a significant difference between the two groups. In contrast to the lateromedial pathway of PSs, the mediolateral trajectory of cortical screws may help to avoid an unintentional canal breach.^16,37^ However, Ding et al. reported medial cortex violations in the CBT method according to the learning curve of the surgeon and recommended the lateral starting point for unskilled surgeons to avoid screw-related complications.^20^ Modification of the entry point to the lateral side of the pars can minimize the medial penetration of the pedicle and prevent screw loosening.

There was no significant difference between screw loosening and screw pullout in the two groups. Even if only two studies were involved, several studies demonstrated higher pullout strength and insertional torque or similar biomechanical strength (bending, and rotation force) in the CBT method compared to PSs.^7-^^9,38,39^ However, some studies reported that the resistance to cyclic loading, pullout strength, and lateral bending force was biomechanically superior in the PS group.^14,15^ Lee et al. recently reported under 20 degrees in the sagittal angle and above 14 degrees in the axial trajectory angle could be the cause of screw loosening because an inaccurate angle does not allow the screw to make sufficient contact with the cortical bone.^40^ Without an interbody appliance in the CBT, lower stiffness in axial loading may cause early screw failure by micromotion.^15,16^ Technical errors in CBT including impingement of the screw head to the base of the spinous process and lamina and intraoperative pars fracture are related to screw loosening because a decreased cephalad angulation of the corridor interferes with broadened cortical bone contact.^41,42^ Since the spondylolysis defect in the pars interarticularis is the cause of decreased insertional torque, the CBT method should be used with caution in elderly patients with spondylolysis.^43^

Fusion rates are one of the most important indicators to confirm the success of the surgery, and there was no statistically significant difference in fusion rates between the two groups. Clinical studies reported that the fusion rate was higher in the PS group due to the lower potential risk of micromotion during axial rotation and lateral bending.^11,12^ In CBT, with an interbody device, the biomechanical stability was comparable to that of PSs.^15,16^ A previous study reported that PSs had superior resistance to that of cyclic loading and higher pullout strength than the CBT method due to the anatomical variation in the lamina, limiting sufficient fixation of the CBT, and the potential risk of damaging the four cortices (pars, inferior and superior cortices of the pedicle isthmus, junction of the superior margin of the pedicle, and superior endplate) by rotating around a fulcrum in the CBT method.^14^

The CBT technique using a medial starting point has benefits in reducing the dissection of the superior facet joint and muscle, minimizing surgical trauma. The CBT method showed significantly lower rates of SFJV. The caudomedial entry point near the pars interarticularis in the CBT contributes to a lower risk of SFJV, which reduces the incidence of adjacent segment disease (ASD).^44^ The incidence of sASD was significantly higher in the PS group due to the lateral starting point. Encroachment of the adjacent facet joint results in increased facet joint contact force and intradiscal pressure above the adjacent segment with extension and torsional motion.^45^ Facet joint arthrosis and intervertebral disc disorder can be the cause of ASD. In addition, excessive posterior decompression and longer transpedicular fusions can endanger adjacent segment stability.^46^ For these reasons, ASD can be the cause of revision surgery. This meta-analysis revealed that sASD was the most common reason for revision surgery in both the CBT and PS groups. The revision rates were significantly lower in the CBT group because the better-preserved facet joint may contribute to the stability of the upper segment (Table 6).

The CBT method was more advantageous in terms of operation time, bleeding amount, incision range, and hospital length of stay. CBT can fix the screw with a smaller incision than the PS method, thereby ensuring less bleeding and faster operative time. Increasing operative duration is an independent risk factor for lumbar fusion and can increase the risk of postoperative complications^47^ such as pulmonary thromboembolism, infection, and venous thromboembolism.^48^

The CBT method has the advantage of enhanced fixation in osteoporotic bones; less surgery-related morbidity (operation time, estimated blood loss, and length of stay); decreased SFJV, sASD, and revision rates, and the overall incidence of complications. The SFJV and sASD are important factors in determining patient prognosis after surgery and the CBT method showed lower SFJV and sASD incidences. However, there were not enough enrolled studies to demonstrate statistical significance for SFJV and sASD. Although many studies demonstrated that the CBT method was biomechanically superior or equal to PSs, most of the studies were conducted in vitro and there were not enough in vivo CBT method results to demonstrate the replaceability of PSs.

This meta-analysis revealed that CBT was related to lower surgical morbidities, lower revision rates, and lower overall complication rates including sASD and SFJV. However, both the CBT and PS methods had similar fusion rates and complications, including hardware complications and wound infections. The CBT method did not outperform PSs in all respects. Therefore, selecting a surgical method in consideration of the patient’s BMD, the condition of the pars interarticularis, or the skill level of the surgeon is recommended. Prognostic evaluation through long-term follow-up is required. More high-quality RCTs are required to verify and strengthen our results.

This meta-study had several limitations. First, only two studies were RCTs, both with fewer than 50 patients in each arm. Of the remaining 14 studies, all were cohort studies with only three studies having more than 50 patients in each arm, which tended to show exaggerated outcomes. Second, only seven studies had two or more years of follow-up. Third, the included studies did not reflect the outcomes according to the learning curve, which is a potential cause of the heterogeneous outcomes.

**Highlights**

- The cortical bone trajectory (CBT) technique showed lower surgical morbidities, lower revision rates, and lower overall complication rates including symptomatic adjacent disease (sASD) and superior facet joint violation (SFJV) than the pedicle screw (PS) method in this meta-analysis.
- However, the PS technique showed fusion rates and incidence of hardware complications and wound infections similar to those of the CBT method.
- The CBT method did not surpass PSs in all aspects.
- Therefore, selecting a surgical method in consideration of the patient`s BMD, the condition of the pars interarticularis, or the skill level of the surgeon is recommended.
- A new cortical bone trajectory (CBT) technique has been proposed as an alternative to pedicle screw fixation method for posterior lumbar fusion.

## Figures and Tables

**Table 1.** Study Characteristics

	Study design	No. of patients		Mean age		Sex (M/F)		Follow-up (months)		Fusion technique	Fixation technique		Country
		CBT	PS	CBT	PS	CBT	PS	CBT	PS	ss	CBT	PS	
Chin et al.^25^(2017)	Cohort	30	30	48 ± 3	62 ± 3	18/12	15/15	24	24	NM	1.5 inch midline incision	open traditional	USA
Chen et al.^22^(2016)	Cohort	18	15	53.39 ± 1.97	59.2 ± 3.12	11/7	2/13	15	15	NM	minimal incision	open traditional	USA
Hoffman et al.^32^(2019)	Cohort	25	23	53.4	48.5	16	16	52.5	52.5	MIDLF(CBT), TLIF(PS)	MIDLF minimal incision	open traditional	USA
Hung et al.^23^(2016)	Cohort	16	16	60.37 ± 11.07	64.12 ± 5.79	5/11	6/10	18	18	PLIF	open	open traditional	China
Lee & Shin^29^ (2018)	RCT	22	31	51.2 ± 11.9	51.7 ± 10.4	31/4	33/4	24	24	PLIF	open	open traditional	Korea
Lee et al.^11^(2015)	RCT	38	39	51.3 ± 12.4	51.9 ± 11.7	33/5	34/5	12	12	PLIF	open	open traditional	Korea
Lee & Ahn^28^ (2018)	Cohort	35	37	32.7 ± 10.1	64.2 ± 9.3	9/13	12/19	12	12	PLIF + PLF	open	open traditional	Korea
Liu et al.^33^(2019)	Cohort	50	54	68 ± 5	fa	26/24	27/27	36	36	PLIF	open	open traditional	China
Malcolm et al.^30^(2018)	Cohort	45	35	63 ± 9	57 ± 11	20/25	7/28	12	12	TLIF	open	open traditional	USA
Marengo et al.^31^(2018)	Cohort	18	17	45 ± 9.63	54 ± 12.01	12/8	9/11	12	12	PLIF	open	open traditional	Italy
Peng et al.^26^(2017)	Cohort	51	46	62.8	61.9	23/28	21/25	24	24	PLIF	open	open traditional	China
Sakaura et al.^13^(2016)	Cohort	95	82	68.7 ± 9.5	67 ± 8.7	46/49	36/46	35	40	PLIF	open	open traditional	Japan
Sakaura et al.^12^(2018)	Cohort	22	20	70.7 ± 7.3	68.3 ± 9.6	4/18	6/14	39	35	PLIF	open	open traditional	Japan
Sakaura et al.^33^(2019)	Cohort	102	77	67.5 ± 9.2	66.4 ± 10.5	35/67	28/49	36	36	PLIF	open	open traditional	Japan
Takenaka et al.^14^(2017)	Cohort	42	77	65.7 ± 8.1	65.7 ± 11.4	18/24	31/46	17	35	PLIF	open	open traditional	Japan

CBT: cortical bone trajectory, PS: pedicle screw, PLIF: posterior lumbar interbody fusion, TLIF: transforaminal lumbar interbody fusions, PLF: posterolateral lumbar fusion, MIDLF: midline lumbar fusion.

**Table 2.** Various Outcomes between CBT and PS Groups in the Included Studies

	Complications										Surgical practicability											
	Overall complication No./%		Hardware complications No.(Total)		Superior facet joint violation No.(Total)		Symptomatic ASD No.(Total)		Wound infection No.(Total)		Fusion rate. No./%		Time (min.)		Intraoperative blood loss (mL)		Incision length (cm)		Length of hospital stay (days)		Revision surgery No./%	
Authors (year)	CBT	PS	CBT	PS	CBT	PS	CBT	PS	CBT	PS	CBT	PS	CBT	PS	CBT	PS	CBT	PS	CBT	PS	CBT	PS
Chin et al.^25^ (2017)											29/96.6	27/90	138 ± 10	254 ± 24	152 ± 28	319 ± 87			3.6 ± 1.7	4.6 ± 2.3		
Chen et al.^22^ (2016)											18/100	15/100										
Hoffman et al.^32^ (2019)	2/16.6	3/13.0	2(25)	3(23)											186 ± 114	416 ± 290	3.6 ± 1.7	4.6 ± 2.3			0/0	1/4.3
Hung et al.^23^ (2016)											16/100	16/100	237 ± 63	265 ± 85	218 ± 78	272 ± 78	5.1 ± 1.4	5.8 ± 0.5	5.1 ± 1.4	5.8 ± 0.5		
Lee & Shin^29^ (2018)											20/90.9	28/90.3	78 ± 18	156 ± 48	231 ± 78	593 ± 93	4.5 ± 1.1	6.9 ± 0.9	4.5 ± 1.1	6.9 ± 0.9		
Lee et al.^11^ (2015)	0/0.0	10/25.6	0(38)	2(39)	0 (38)	7 (39)			0(38)	1(39)	35/92.1	34/87.1	156 ± 12	126 ± 18	360 ± 30	450 ± 25	13.7 ± 2.2	13.8 ± 1.7	13.7 ± 2.2	13.8 ± 1.7		
Lee & Ahn^28^ (2018)	10/28.5	17/45.9	6(35)	9(37)			4(35)	8(37)			33/94.2	35/94.6										
Liu et al.^33^ (2019)	1/2.0	3/5.5	1(50)	2(54)					0(50)	1(54)			223 ± 17	221 ± 15	166 ± 13	226 ± 12	8.1 ± 1.8	11.1 ± 2.1	8.1 ± 1.8	11.1 ± 2.1		
Malcolm et al.^30^ (2018)	1/2.2	2/6.5		1(35)					1(45)	1(35)	45/100	35/100	214 ± 6	262 ± 71	231 ± 186	424 ± 315	4.3 ± 1.6	4.8 ± 1.7	4.3 ± 1.6	4.8 ± 1.7	1/2.2	1/2.8
Marengo et al.^31^ (2018)	5/25.0	11/55.0	3(20)	3(20)	1(20)	7(20)			1(20)	1(20)	18/90.0	17/85.0	157 ± 21	169 ± 23	276 ± 67	330 ± 90	2.9 ± 1.3	3.8 ± 1.3	2.9 ± 1.3	3.8 ± 1.3		
Peng et al.^26^ (2017)	1/1.9	2/4.3							1(51)	2(46)			117 ± 24	152 ± 36	121 ± 43	212 ± 65	4.7 ± 1.8	7.3 ± 3.4	4.7 ± 1.8	7.3 ± 3.4		
Sakaura et al.^13^ (2016)	8/8.4	13/15.8	2(95)	3(82)			3(95)	9(82)	2(95)	1(82)	84/88.4	79/96.3	123 ± 24	145 ± 33	205 ± 152	204 ± 145					6/6.3	12/14.6
Sakaura et al.^12^ (2018)	3/13.6	7/35.0	0(22)	1(20)			2(22)	4(20)	1(22)	1(20)	40/91.0	38/95.0	192 ± 30	218 ± 49	495 ± 386	612 ± 424					0/0	2/10
Sakaura et al.^33^ (2019)	1/0.9	3/3.8					1(102)	3(77)													1/0.9	3/3.9
Takenaka el al.^14^ (2017)	0/0.0	2/2.6							0(42)	2(77)	34/28.6	63/25.9	120 ± 15	125 ± 25	119 ± 76	204 ± 117					1/1.6	2/2.1

* not mentioned. CBT: cortical bone trajectory, PS: pedicle screw, ASD: adjacent segment disease, JOA: Japanese orthopedic association, ODI: Oswestry disability index, VAS: visual analog scale.

**Table 3.** Indications for CBT Method

Chin et al.^25^ (2017)	Lumbar disk herniation, Degenerative disk disease Spinal stenosis, Radiculopathy, Spondylolisthesis
Chen et al.^22^ (2016)	Lumbar degenerative disease, Lumbar instability
Hoffman et al.^32^ (2019)	Lumbar degenerative disease
Hung et al.^23^ (2016)	Spondylosis with spinal stenosis Degenerative spondylolisthesis
Lee & Shin^29^ (2018)	Adjacent segmental disease
Lee et al.^11^ (2015)	Spinal stenosis with foraminal stenosis Isthmic spondylolisthesis
Lee & Ahn^28^ (2018)	Spinal stenosis, Spondylolisthesis
Liu et al.^33^ (2019)	Spinal stenosis, Spondylolisthesis
Malcolm et al.^30^ (2018)	Spinal stenosis, Spondylolisthesis
Marengo et al.^31^ (2018)	Spinal stenosis (foraminal type) Disk herniation with discopathy, Spondylolisthesis
Peng et al.^26^ (2017)	Spinal stenosis, Spondylolisthesis, Lumbar instability
Sakaura et al.^13^ (2016)	Spondylolisthesis
Sakaura et al.^12^ (2018)	Spondylolisthesis
Sakaura et al.^33^ (2019)	Spondylolisthesis
Takenaka el al.^14^ (2017)	Spondylolisthesis (isthmic type), Spinal stenosis (foraminal type) Disk herniation

**Table 4.** Newcastle-Ottawa Scale

Author	Level of evidence	Selection	Comparability	Outcomes	Quality judgment
Chin et al.^25^ (2017)	III	4	1	3	8
Chen et al.^22^ (2016)	IV	4	1	2	7
Hoffman et al.^32^ (2019)	IV	4	2	2	8
Hung et al.^23^ (2016)	IV	4	2	2	8
Lee & Ahn.^28^ (2018)	III	4	2	2	8
Liu et al.^33^ (2019)	III	4	2	2	8
Malcom et al.^30^ (2018)	III	4	2	2	8
Marengo et al.^31^ (2018)	III	4	2	2	8
Peng et al.^26^ (2017)	IV	4	1	2	7
Sakaura et al.^13^ (2016)	III	4	2	3	9
Sakaura et al.^12^ (2018)	III	4	2	3	9
Sakaura et al.^33^ (2019)	III	4	2	3	9
Takenaka el al.^14^ (2017)	III	4	1	3	8

**Table 5.** Types of Hardware Complications

Authors (year)	CBT (No.)	PS (No.)
Hoffman et al.^32^ (2019)	Screw loosening (1) Screw pullout (1)	Screw loosening (1) Screw pullout (1) Screw malposition (1)
Lee et al.^11^ (2015)	(0)	Screw malposition (2)
Lee & Ahn^28^ (2018)	Screw loosening (4) Cage subsidence (2)	Screw loosening (7) Cage subsidence (2)
Liu et al.^33^ (2019)	Cage migration (1)	Screw pullout (2)
Marengo et al.^31^ (2018)	Screw malposition (3)	Screw malposition (3)
Sakaura et al.^13^ (2016)	Screw malposition (2)	Screw malposition (3)
Sakaura et al.^12^ (2018)	(0)	Screw malposition (1)

CBT: cortical bone trajectory, PS: pedicle screw.

**Table 6.** Reasons for Revision

Authors (year)	CBT (No.)	PS (No.)
Hoffman et al.^32^ (2019)	(0)	Screw malposition (1)
Malcolm et al.^30^ (2018)	sASD (1)	sASD (1)
Sakaura et al.^13^ (2016)	Screw malposition (2) sASD (3)	Screw malposition (3) sASD (8)
Sakaura et al.^12^ (2018)	(0)	sASD (2)
Sakaura et al.^33^ (2019)	sASD (1)	sASD (3)
Takenaka el al.^14^ (2017)	(0)	Deep wound infection (2)

CBT: cortical bone trajectory, PS: pedicle screw, sASD: symptomatic adjacent segment disease.
